# More Tweets, More Votes: Social Media as a Quantitative Indicator of Political Behavior

**DOI:** 10.1371/journal.pone.0079449

**Published:** 2013-11-27

**Authors:** Joseph DiGrazia, Karissa McKelvey, Johan Bollen, Fabio Rojas

**Affiliations:** 1 Department of Sociology, Indiana University, Bloomington, Indiana, United States of America; 2 School of Informatics and Computing, Indiana University, Bloomington, Indiana, United States of America; CSIC-Univ Miguel Hernandez, Spain

## Abstract

Is social media a valid indicator of political behavior? There is considerable debate about the validity of data extracted from social media for studying offline behavior. To address this issue, we show that there is a statistically significant association between tweets that mention a candidate for the U.S. House of Representatives and his or her subsequent electoral performance. We demonstrate this result with an analysis of 542,969 tweets mentioning candidates selected from a random sample of 3,570,054,618, as well as Federal Election Commission data from 795 competitive races in the 2010 and 2012 U.S. congressional elections. This finding persists even when controlling for incumbency, district partisanship, media coverage of the race, time, and demographic variables such as the district's racial and gender composition. Our findings show that reliable data about political behavior can be extracted from social media.

## Introduction

An increasingly important question for researchers in a variety of fields is whether social media activity can be used to assess offline political behavior. Online social networking environments present a tremendous scientific opportunity: they generate large-scale data about the communication patterns and preferences of hundreds of millions of individuals [1], which can be analyzed to form sophisticated models of individual and group behavior [2], [3]. However, some researchers have questioned the validity of such data, pointing out that social media content is largely focused on entertainment and emotional expression [4], [5], potentially rendering it a poor measure of the behaviors and outcomes typically of interest to social scientists.

Additionally, social media provide a self-selected sample of the electorate. A study by Mislove et al. investigates this bias on the county level, finding that Twitter data do not accurately represent the sociodemographic makeup of the United States [6]. Furthermore, right-leaning political communication channels, such as #tcot (“Top Conservatives on Twitter”), are more active and densely connected than left-leaning channels [7]. Hargittai's work has been extremely influential in investigating gender, income, age, and other social factors that create systematic differences in Internet use, including Twitter [8]–[10]. Researchers have also found that extraversion and openness to experiences are positively related to social media use, while emotional stability has a negative relationship [11]. Taken together, these studies suggest that social media provide a biased, non-representative sample of the population.

Despite these issues, a growing literature suggests that online communication can still be a valid indicator of offline behavior. For example, film title mentions correlate with box office revenue [12], and online expressions of public mood correlate with fluctuations in stock market prices, sleep, work, and happiness [13]–[15]. In addition, a number of studies have examined the relationship between online activity and election outcomes [16]–[19]. However, many of these studies have been criticized for a variety of reasons, including: using a self-selected and biased sample of the population; investigating only a small number of elections; or not using sociodemographic controls [20], [21]. Tumasjan analyzed the relationship between tweets and votes in the 2009 German election [18], but these results have been criticized because they depend upon arbitrary choices made by the authors in their analysis [22].

Here, we provide a systematic link between social media data and real-world political behavior. Over two U.S. congressional election cycles, we show a statistically significant relationship between tweets and electoral outcomes that persists after accounting for an array of potentially confounding variables, including incumbency, baseline district partisanship, conventional media coverage and the sociodemographics of each district. These results do not rely on any knowledge of the physical location of these users at the time of their post or their emotional valence. These results indicate that the “buzz” or public discussion about a candidate on social media can be used as an indicator of voter behavior.

## Materials and Methods

### Data

To model the relationship between social media content and political behavior in a manner that overcomes the limitations of previous work, we compiled a congressional district-level dataset with data from Twitter, the Federal Election Commission, and the U.S. Census Bureau. First, we retrieved a random sample of 547,231,508 tweets posted between August 1 and November 1, 2010 and 3,032,823,110 posted between August 1 and November 5, 2012. We collected this data using the Twitter “Gardenhose” streaming API (https://dev.twitter.com/docs/api/1.1/get/statuses/sample), which provides a random sample of approximately 10% of the entire Twitter stream. Data on tweets collected through the API include useful metadata, including a unique tweet identifier, the content of the tweet, a timestamp, and the username of the account that produced the tweet. Of this random sample, we extracted 113,985 tweets in 2010 and 428,984 in 2012 that contained the name of the Republican or Democratic candidate for Congress from each district. We have released this district-level data online (http://dx.doi.org/10.7910/DVN/23103).

Next, we collected data on election outcomes from the 2010 and 2012 U.S. congressional elections from the Federal Election Commission. Additionally, for 2010, we collected socio-demographic and electoral control variables commonly used in other research on electoral politics for all 435 U.S. House districts [23], [24]. These include measures of Republican incumbency, district partisanship, median age, percent white, percent college educated, median household income and percent female [25]–[27]. Incumbency is coded as 1 if the Republican candidate is an incumbent and 0 otherwise. District partisanship is measured by the percentage of the 2008 presidential vote captured by Republican candidate John McCain. The inclusion of controls is necessary to ensure that the effect of Twitter is robust and not spurious. For example, it is possible that an observed relationship between Twitter mentions and vote counts is simply due to the tendency of Twitter users to discuss incumbents, who have a high probability of winning, more than they discuss non-incumbents. Equivalent data about the sociodemographic profile of congressional districts is not yet available for the 2012 House districts due to redistricting after the 2010 elections.

To control for the extent to which a candidate is covered in the traditional media, we have included a measure of how frequently a candidate is mentioned in transcripts of broadcasts on the cable news network CNN during the same time period. The CNN variable consists of the share of CNN transcripts that mention the Republican candidate. For each district, 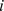, the CNN share variable, 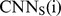, is defined as 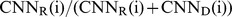 where 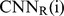 represents the number of transcripts mentioning the Republican candidate and 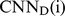 represents the number of transcripts mentioning the Democratic candidate.

### Variable Definitions

Our independent variables are constructed from the number of tweets that contained the candidate's name (e.g. “Nancy Pelosi”). In the formula below, 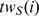 represents the percentage of Twitter attention given to a particular candidate over his or her opponent in a particular race. Each district 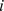 is assigned its share of Republican tweets from the total of both Democratic and Republican frequencies, denoted 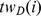 and 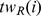 respectively.
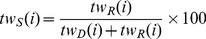
(1)


We construct a similar Twitter share variable to account for the number of users with at least one tweet about a candidate. This totaled 28,193 users in 2010 and 166,978 users in 2012, though the 2012 value is inflated somewhat by vice-presidential candidate, Paul Ryan. We hypothesize this may help account for the potential bias that could be created by a small number of extremely committed users or automated accounts generating large numbers of tweets about a candidate. Tweet share and user share each range from 0 to 100 with tweet share having a median of 50.24 in 2010 and 54.05 in 2012 and user share having a 2010 median of 50.

Our dependent variable consists of the Republican vote share for each district 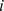, denoted 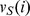 defined as the share of votes received by the Republican candidate, denoted 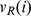, and the Democratic candidate, denoted 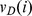.
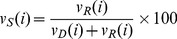
(2)


### Analysis

We estimate the effect of Twitter activity on electoral outcomes using three ordinary least squares regression (OLS) models. We did not use data from 29 districts in 2010 and 46 districts in 2012 where there was no opposition from a major party candidate. For the 2010 data, we estimate bivariate models and full models, which include the aforementioned control variables, for both tweet and user share. For 2012, we only estimate the effect of tweet share on electoral outcomes in a bivariate model, as control variables are not available at the time of publication.

## Results

**Figure 1:**
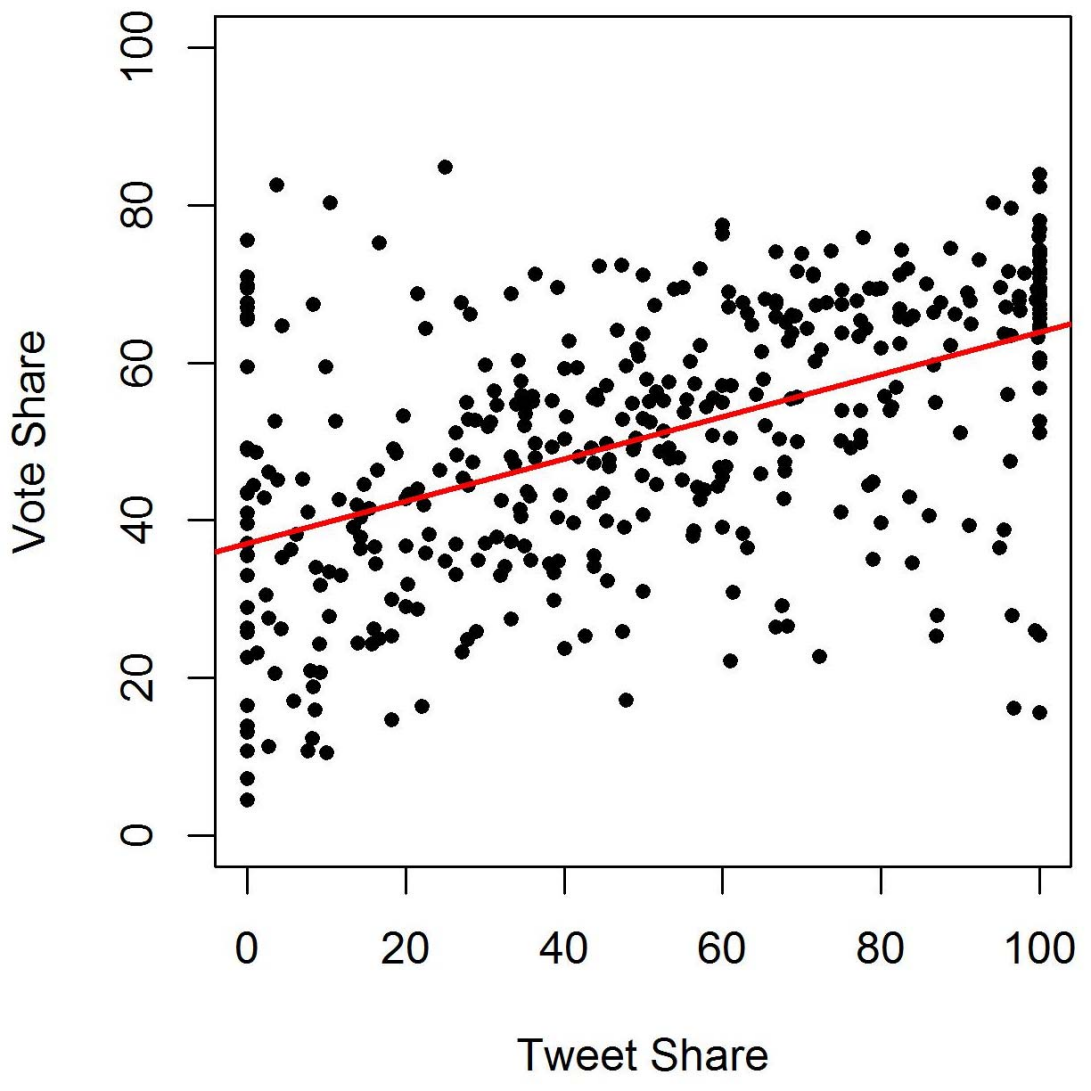
2010 Republican Tweet Share vs. Vote Share. Bivariate relationship between the share of occurrences of Republican names in tweets and vote share in the 2010 congressional elections. We show a significant positive relationship.

Tables 1 and 2 report results from the data from the 2010 election. The coefficients for both the tweet and user share show statistically significant effects (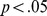). The 2010 bivariate relationship is shown in Figure 1. As shown in Table 1, each percentage point increase in 2010 tweet share is associated with an increase in the vote share of .268 in the bivariate model. Although the effect size is reduced to .022 in the full model, the effect remains highly significant. Both the bivariate and full models fit the data well; the 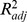 for the bivariate model is .26 and increases to .92 in the full model. The effect for user share is .279 in the bivariate model and .027 in the full model, indicating that, net of all other factors, each additional percentage point increase in user share is associated with an increase of .027 in the Republican vote share. The effect of user share is also significant, indicating that this relationship is not driven by a small number of users. Like the tweet share models, both the bivariate and full user share models fit the data well with 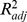 values of .28 and .92, respectively.

**Table 1:** Results for Regression of Republican Vote Share on Tweet Share with Controls.

Variable	Bivariate (SE)	Full Model (SE)
Republican Tweet Share	0.268 (0.022)***	0.022 (0.01)*
Republican Incumbent		11.06 (0.66)***
% McCain		0.776 (0.03)***
Median Age		0.012 (0.09)
% White		0.129 (0.02)***
% College Educated		−0.004 (0.05)
Median HH Income		0.016 (0.03)
% Female		0.089 (0.30)
CNN share		0.002 (0.01)
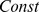	37.042 (1.35)	−4.07 (15.04)
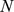	406	406
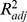	.26	.92

Explaining Republican vote share with the proportion of tweets that included a Republican candidate during the three months before the 2010 election. The share of Republican tweets remains significant after adding controls. Standard error (SE) is in parentheses.

* (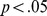).

** (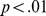).

*** (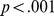).

**Table 2:** Results for Regression of Republican Vote share on User Share with Controls.

Variable	Bivariate (SE)	Full Model (SE)
Republican User Share	0.279 (0.02)***	0.027 (0.01)**
Republican Incumbent		10.956 (0.65)***
% McCain		0.772 (0.03)***
Median Age		0.010 (0.09)
% White		0.131 (0.02)***
% College Educated		−0.005 (0.05)
Median HH Income		0.017 (0.03)
% Female		0.117 (0.30)
CNN share		0.001 (0.01)
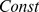	36.423 (1.32)	−5.474 (15.01)
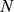	406	406
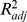	.28	.92

Explaining Republican vote share with the proportion of users who included a Republican candidate in at least one tweet during the three months before the 2010 election. The relationship remains significant after adding controls. Standard error (SE) is in parentheses.

* (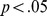).

** (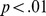).

*** (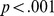).

To give a better sense of the magnitude of the effects, an increase of one standard deviation in tweet share in the full model is associated with an increase in the vote share equal to .708. An increase of one standard deviation in user share is associated with an increase of .874 in vote share in the full model. While these effects are much smaller than the effects of the Twitter measures in the bivariate model, modest increases in the tweet share measures still produce substantively meaningful and highly significant predicted changes in the vote share.

There are also a number of significant effects for some of the other control variables that are worth noting. Consistent with previous research, Republican incumbency and baseline district partisanship, as measured by McCain vote share, have highly significant effects in both models [23], [24]. Interestingly, the percentage of whites also has a highly significant positive effect on vote share, even controlling for McCain vote share. This may indicate that voting in this election was particularly racialized even compared to the 2008 contest.

We can assess the limitations of this model by looking at outliers. We examine those congressional districts where the residual was at least two standard deviations above or below the predicted value. We find that districts where the model under-performs tend to be relatively noncompetitive. If there is little doubt about who the winner will be, there may be little reason to talk about the election. In the baseline model, for example, we obtain outliers such as California's 5th Congressional District and West Virginia 2nd Congressional District. These areas lean heavily Democratic. California's 5th has voted Democrat since 1949. Since 2000, every Democrat polled at least 70%, with the exception of a 2005 special election, where the winning Democrat won with 67% of the vote. Similarly, West Virginia's 2nd shows a strong partisan orientation. A single Republican has held the seat since 2001. However, a lack of competition does not explain every outlier. Some districts have idiosyncratic features that merit more research. For example, Oklahoma's 2nd Congressional District is a rural area that has voted for a Democratic Congressman while voting strongly for McCain and Bush.

**Figure 2:**
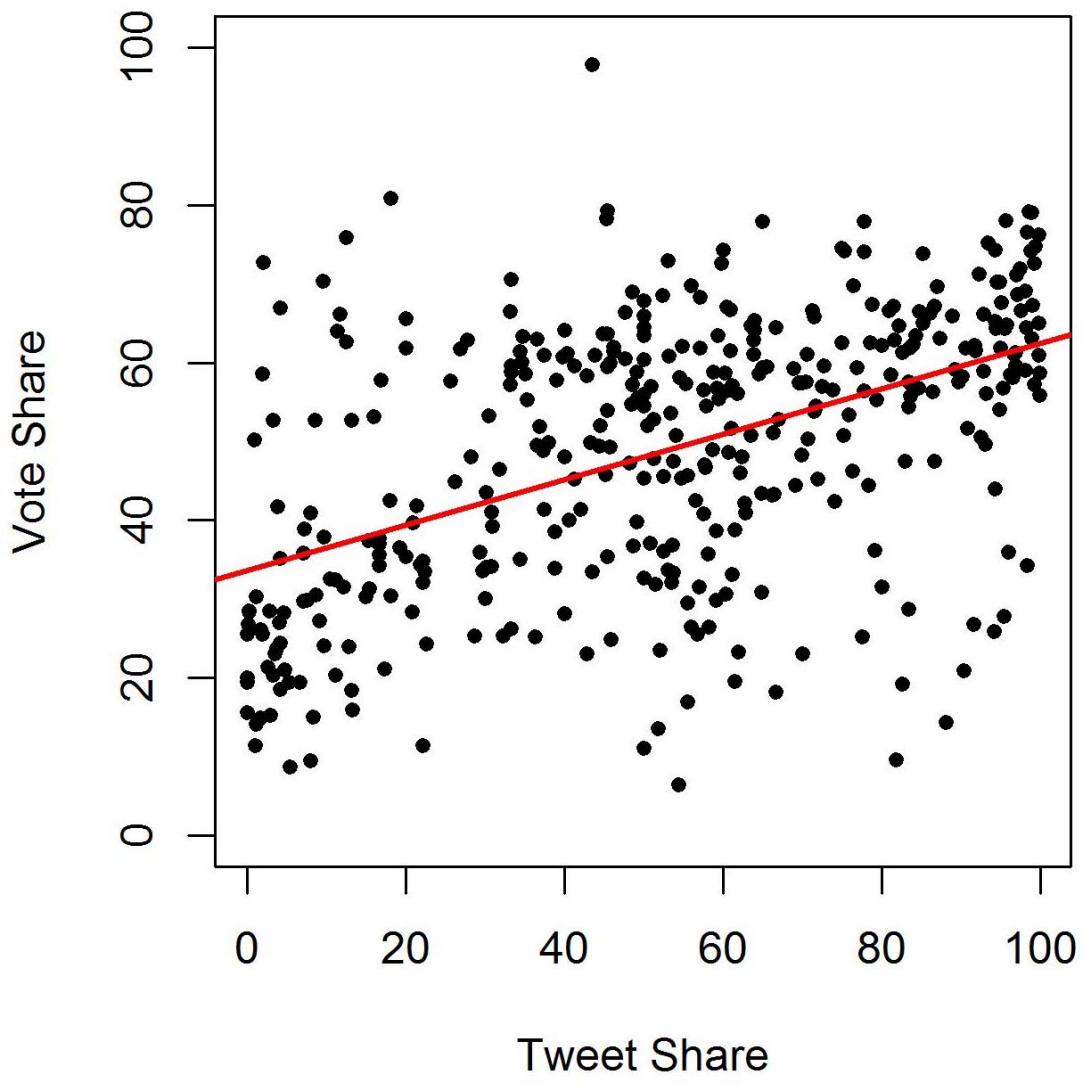
2012 Republican Tweet Share vs. Vote Share. Bivariate relationship between the share of occurrences of Republican names in tweets and vote share in the 2012 congressional elections. We show a significant positive relationship.

The analysis of the 2012 U.S. House elections yields similar bivariate results. The bivariate relationship between tweet and vote share is shown in Figure 2 for 2012. Data from 389 districts with competitive races yields a bi-variate OLS regression coefficient of .288 (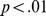). We observe an analogous effect, with very similar coefficients in the bivariate models across the two election cycles.

**Figure 3:**
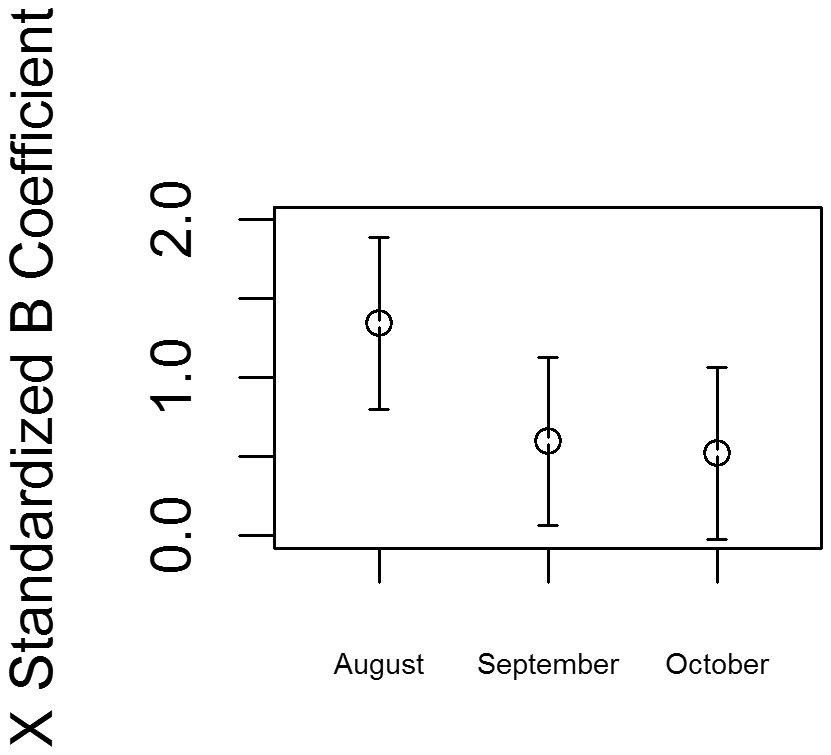
Effects of Name Share Mention by Month. Effects of Republican tweet share during the months of August, September, and October with a 95% confidence interval.

Finally, we test the robustness of the results by examining the model across different time periods before the election. Because the link between tweets and voter preferences may vary during the period before the election, we estimate the same models using only monthly shares of Twitter data from August, September, and October. As shown in Figure 3, the effect of Twitter share is similar in magnitude across all three months as indicated by their overlapping confidence intervals.

## Discussion

These findings indicate that the amount of attention received by a candidate on Twitter, relative to his or her opponent, is a statistically significant indicator of vote share in 795 elections during two full election cycles. Note that this is found in a random sample of all tweets during the first three months before the two election cycles, despite the fact that Twitter has been well-studied as a biased sample of the general population [6]. Our analysis does not require information about the physical location of Twitter users. Further research can investigate why geographical information about users is not needed. Furthermore, we find that social media are a better indicator of political behavior than traditional television media, such as CNN, which many scholars have argued is important because it shapes political reality via agenda setting [28], [29].

The effect of Twitter holds even without accounting for the sentiment of the tweet – in other words, it holds regardless of whether or not the tweet is positive or negative (e.g., “I love Nancy Pelosi,” “Nancy Pelosi should be impeached”). One possible explanation draws on previous research in psycho-linguistics, which has found that people are more likely to say a word when it has a positive connotation in their mind [30]–[32]. Known as the Pollyana hypothesis, this finding implies that the relative over-representation of a word within a corpus of text may indicate that it signifies something that is viewed in a relatively positive manner. Another possible explanation might be that strong candidates attract more attention from both supporters and opponents. Specifically, individuals may be more likely to attack or discuss disliked candidates who are perceived as being strong or as having a high likelihood of winning.

The findings also suggest that social media data could be developed into measures of public attitudes and behaviors that could serve as alternatives to polling data. While polling data remains extremely useful, and has seen increased public interest with the rise of popular polling analysts like Nate Silver, alternate data sources can serve as an important supplement to traditional voter surveys. This is particularly true in cases like U.S. House races, where large amounts of traditional polling data are typically not available. Social media data has other distinct advantages, including the fact that, because social media data is constantly created in real time, data about particular events or time periods can be collected after the fact. Additionally, social media data is less likely to be affected by social desirability bias than polling data [33]. That is, a person who participates in a poll may not express opinions perceived to be embarrassing or offensive. For example, few survey respondents will admit that may not vote for a candidate because he is Black (e.g., Barack Obama) or a Mormon (e.g., Mitt Romney). The potential of social media and Internet data for capturing these socially undesirable sentiments was demonstrated in recent research on Google searches which showed that the frequency of searches for racial slurs is correlated with a lower vote count for Obama in 2008 relative to Kerry in 2004 [34]. This finding would not be possible with a traditional poll.

Finally, this study adds to the mounting evidence that online social networks are not ephemeral, spam-ridden sources of information. Rather, social media activity provides a valid indicator of political decision making.
